# Emerging Role of DREAM in Healthy Brain and Neurological Diseases

**DOI:** 10.3390/ijms24119177

**Published:** 2023-05-24

**Authors:** Pasquale Molinaro, Luca Sanguigno, Antonella Casamassa, Valeria Valsecchi, Rossana Sirabella, Giuseppe Pignataro, Lucio Annunziato, Luigi Formisano

**Affiliations:** 1 Division of Pharmacology, Department of Neuroscience, Reproductive and Dentistry Sciences, School of Medicine, Federico II University of Naples, Via Pansini, 5, 80131 Naples, Italy; pmolinar@unina.it (P.M.); lucasanguigno@libero.it (L.S.); valsecchiv@yahoo.com (V.V.); sirabell@unina.it (R.S.); gpignata@unina.it (G.P.); 2 IRCCS Synlab SDN S.p.A., Via Gianturco 113, 80143 Naples, Italy; antonellacasamassa@gmail.com

**Keywords:** DREAM, calsenilin, KCNIP3, neurodegeneration

## Abstract

The downstream regulatory element antagonist modulator (DREAM) is a multifunctional Ca^2+^-sensitive protein exerting a dual mechanism of action to regulate several Ca^2+^-dependent processes. Upon sumoylation, DREAM enters in nucleus where it downregulates the expression of several genes provided with a consensus sequence named dream regulatory element (DRE). On the other hand, DREAM could also directly modulate the activity or the localization of several cytosolic and plasma membrane proteins. In this review, we summarize recent advances in the knowledge of DREAM dysregulation and DREAM-dependent epigenetic remodeling as a central mechanism in the progression of several diseases affecting central nervous system, including stroke, Alzheimer’s and Huntington’s diseases, amyotrophic lateral sclerosis, and neuropathic pain. Interestingly, DREAM seems to exert a common detrimental role in these diseases by inhibiting the transcription of several neuroprotective genes, including the sodium/calcium exchanger isoform 3 (NCX3), brain-derived neurotrophic factor (BDNF), pro-dynorphin, and c-fos. These findings lead to the concept that DREAM might represent a pharmacological target to ameliorate symptoms and reduce neurodegenerative processes in several pathological conditions affecting central nervous system.

## 1. Introduction

The downstream regulatory element antagonist modulator (DREAM), also named calsenilin or potassium voltage-gated channel interacting protein 3 (KCNIP3 or KChIP3), is a multifunctional protein of 256 amino acids belonging to EF-hand Ca^2+^ binding protein family. Indeed, DREAM activity is mainly dependent from four EF-hand domains that, upon Ca^2+^ binding, change the protein conformation, and thus its binding ability.

**Figure 1:**
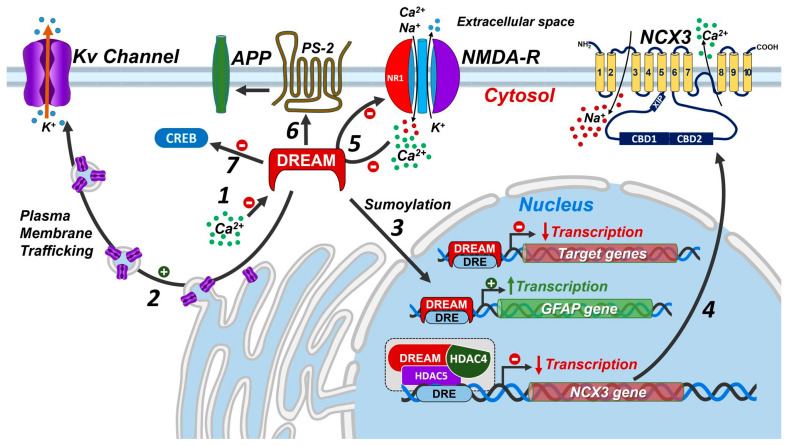
Possible mechanisms of DREAM action. (**1**) High [Ca^2+^] levels inhibits the transcriptional activity of DREAM; (**2**) DREAM participates in the plasma membrane trafficking of the Kv4.2 potassium channel; (**3**) DREAM sumoylation allows entering into the nucleus to exert as transcription and/or epigenetic factor; (**4**) DREAM regulates the transcription of proteins involved in the maintenance of Ca^2+^ homeostasis; (**5**) DREAM regulates the activity of NMDA receptors by directly inhibiting the NR1 subunit; (**6**) DREAM participates in the regulation of presenilin 2 activity; (**7**) DREAM, upon low levels of [Ca^2+^], directly binds to CREB and prevents its phosphorylation and thus its transcriptional activity. Abbreviations: APP, amyloid precursor protein; NMDA-R, N-methyl-D-aspartate receptor; PS-2, presenilin-2; NCX3, Na^+^/Ca^2+^ exchanger 3; GFAP, Glial fibrillary acidic protein.

DREAM was initially identified as an inhibitory transcription factor bound to regulatory elements located downstream from the transcription initiation site of the prodynorphin and c-fos genes [1,2]; however, more recent evidence showed that DREAM exerts different roles in different cell compartments [1,3]. For instance, DREAM can serve as an inhibitory transcription factor in the nucleus but can also directly regulate cytosolic proteins involved in the membrane excitability or calcium homeostasis outside the nucleus (Figure 1). Intriguingly, recent data suggest that the localization of DREAM in the nucleus, and thus its transcriptional activity, might be determined by the sumoylation on lysine residues in position 26 and 90 of this Ca^2+^-binding protein [4].

### 1.1. Control of Gene Expression by DREAM in the Nucleus

**Figure 2:**
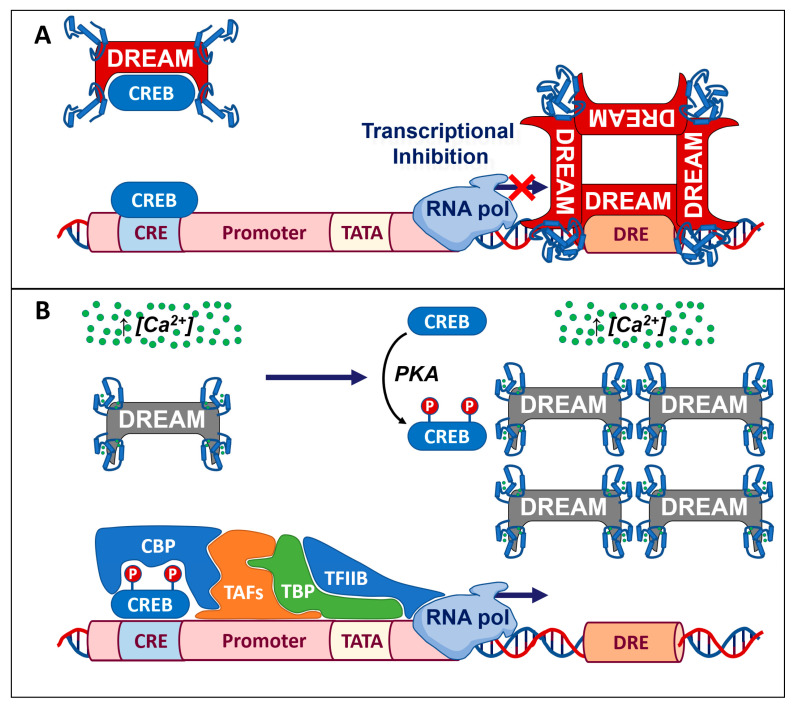
Transcriptional regulations of DREAM. (**A**) Upon low levels of [Ca^2+^]_n_, DREAM forms a tetramer that binds to DRE sequences on the target genes and inhibits their transcription. Furthermore, DREAM directly binds to CREB preventing its PKA-dependent phosphorylation and, thus, its activating transcription. (**B**) Upon the rise in [Ca^2+^]_n_ levels, DREAM is inactivated, allowing for the detachment of this Ca^2+^-sensor protein from DRE sequences and CREB protein. Then, CREB can be phosphorylated and can bind the coactivator CBP, which, in turn, recruits the basal transcription activator factors (TAFs), TATA-binding protein (TBP) and transcription factor TFIIB. The resulting complex stabilizes RNA polymerase II, contributing to the assembly of the transcriptional initiation complex and, ultimately, relieves the transcriptional repression of target genes.

One of the major mechanisms by which DREAM controls gene transcription is the binding to a specific DNA sequence named downstream regulatory element (DRE), whose central core is GTCA. DREs can be present in a promoter region as a single copy or two inverted copies. The effects of these DRE elements on the transcription of target genes depend on several conditions, including the localization downstream or upstream to the TATA box sequence [5], nuclear Ca^2+^ levels ([Ca^2+^]_n_), and post-translational modifications of DREAM. Mainly, the presence of DRE sequences downstream of the TATA box of a gene promoter results in an Ca^2+^-regulated transcriptional repression [4]. For these reasons, it is not surprising that many genes provided with DRE sequences participate in the transcriptional regulation of important proteins activated by [Ca^2+^]_i_ including CREB, CtBP1, nuclear receptors or TTF-1 [6,7,8,9]. Indeed, DREAM serves as a transcriptional Ca^2+^ sensor that, in presence of low nuclear Ca^2+^ concentrations ([Ca^2+^]_n_), forms a tetramer that binds to DRE sequences, causing a transcriptional inhibition of the target gene in neurons (Figure 2A). By contrast, upon elevation in [Ca^2+^]_n_ levels following cell stimulations, DREAM detaches from DRE sequences, thus reducing its inhibitory effect on transcription (Figure 2B). Obviously, either the transfection of dominant-negative forms of DREAM or the disruption of the DRE sequences causes the loss of transcriptional inhibition, and thus, there is an increase in the transcriptional activity as it occurs upon Ca^2+^ stimulation. However, the binding of DREAM to DRE sites can be also determined by a direct interaction with specific nuclear proteins of the cAMP pathway in a Ca^2+^-independent manner [2]. Interestingly, these two Ca^2+^-dependent and Ca^2+^-independent regulatory mechanisms of DREAM, that ultimately reduce the DRE-dependent inhibition of target genes, can occur separately or concomitantly.

Most of the DREAM target genes take part in the regulation of Ca^2+^-dependent processes that include members of the cation/Ca^2+^ exchanger superfamily, such as Na^+^/Ca^2+^ exchanger 3 (NCX3) [10]; ligand- or voltage-gated channels [3]; important regulatory transcription factors, such as c-fos and CREB [6]; enzymes, such as monoglyceride lipase (MGLL) and protease cathepsin L (CTSL); hormones, such as preprodynorphin [5] and thyroglobulin [7]; and neurotrophic factors, such as brain-derived neurotrophic factor (BDNF) [11]. The first identified DREAM target gene was preprodynorphin [5], whose transcriptional inhibition is counteracted by a phosphorylation of the Ca^2+^-sensor protein by protein-kinase A (PKA) [2]. In addition, DREAM also regulates the expression of BDNF [11], another gene involved in pain modulation [12,13,14]. Furthermore, DREAM represses the transcription of the c-fos gene that plays a relevant role in coupling neuronal activity to gene expression and mediates neurophysiological functions, including activity-dependent survival, plasticity, and long-term plasticity (LTP) [15,16]. Intriguingly, this early immediate gene is dysregulated in several models of neuropathological conditions in which the transcriptional repressor DREAM has also been found involved, including stroke [17], amyotrophic lateral sclerosis (ALS) [18], Huntington’s disease (HD) [19] and pain [20].

On the other hand, as the opposite to neurons, DREAM is also known to enhance the transcription of some genes when is activated in astrocytes. For instance, DREAM inhibits c-fos transcription as a consequence of the rise in [Ca^2+^]_n_ following glutamate stimulation in neurons [1]. Furthermore, the stimulation of group I metabotropic glutamate receptors 1 and 5 (mGluR1/5) positively increases the neuronal levels of DREAM [21], thus reinforcing its transcriptional inhibition. By contrast, glutamate stimulation in astrocytes causes the nuclear export of DREAM, thus relieving transcription inhibition of its target genes [22]. Intriguingly, this process seems to depend on the same glutamate receptor, mGluR5, present in neurons but involves a different pathway. In addition, DREAM promotes transcription of glial fibrillary acidic protein (GFAP) in astrocytes by binding two regions of its promoter.

**Figure 3:**
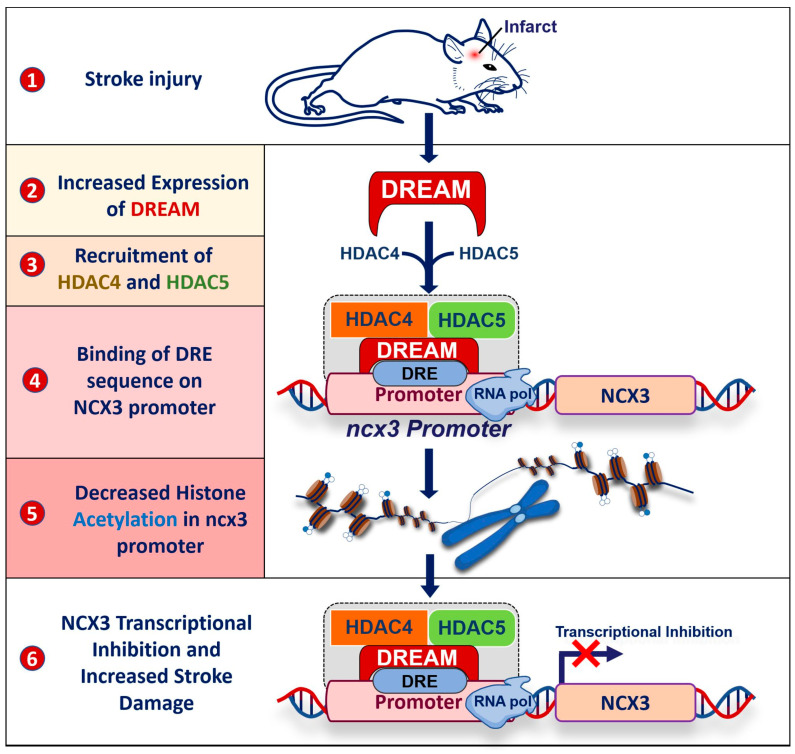
Epigenetic regulation of the gene encoding for Na^+^/Ca^2+^ exchanger isoform 3 (NCX3) following stroke. (**1**) Experimental stroke injury increases DREAM expression in peri-ischemic temporoparietal cortex region (**2**); under these conditions, DREAM recruits the epigenetic enzymes histone deacetylase isoform 4 and 5 (HDAC4 and HDAC5), forming a complex (**3**) that binds to the DRE sequence on ncx3 gene promoter (**4**); this complex deacetylates lysins of histones on ncx3 promoter and inhibits the expression of ncx3 gene (**5**); this process ultimately increases neuronal damage following stroke (**6**).

In addition to the transcriptional activity of DREAM, more recent evidence showed that DREAM can also recruit epigenetic factors in the nucleus forming a complex that downregulates the expression of target genes. For instance, DREAM recruits histone deacetylase isoform 4 and 5 (HDAC4 and HDAC5) enzymes, leading to the deacetylation of histone H4 on the promoter of NCX3 gene after stroke (Figure 3) [23]. This is of particular interest since the downregulation of NCX3 expression during ischemic injury increases brain damage [24,25]. Accordingly, the reduced NCX3 expression levels, either by enhancing DREAM activity or by genetic knock-out of the antiporter, increases neuronal vulnerability to intracellular Ca^2+^ overload and cell death in both in vitro and in vivo models mimicking stroke [10,24]. For these reasons, drugs blocking DREAM activity and/or enhancing NCX3 activity might constitute an innovative therapeutic strategy in stroke [26].

Based on these extensive and complex regulatory mechanisms of DREAM in the control of Ca^2+^-related processes, it is not surprising that this Ca^2+^-binding protein supervises the on/off status of specific Ca^2+^-dependent programs that control synaptic plasticity, learning, memory and neuronal death [27,28]. On the other hand, DREAM could also participate in neurodegenerative diseases where there is a dysregulation of Ca^2+^ homeostasis.

### 1.2. Control of Protein Activity by DREAM Outside the Nucleus

Interestingly, DREAM was initially identified by three independent research groups since it shows several seemingly unrelated mechanisms of action. Indeed, besides the transcriptional activity in nucleus, DREAM exerts a significative binding activity with several proteins in the cytosolic compartment [3,29]. In fact, by leveraging a yeast two-hybrid screening approach, DREAM was identified as a new unknown protein interacting with the carboxy terminal region of presenilin-2 [29] and, for these reasons, was called calsenilin. Although this interaction was not further investigated, later, by using a similar approach, DREAM was erroneously identified as a new protein interacting with the amino terminal domain of Kv4.2 potassium channel [3] and, for these reasons, was also called potassium channel interacting protein (KchIP). In this case, DREAM directly interferes with the potassium channel properties and, thus, its currents depend on [Ca^2+^] levels and the activation of cAMP pathway. On the other hand, details of these mechanisms are still unknown because of the pleiotropic activities of this Ca^2+^-binding protein. Indeed, DREAM, upon phosphorylation of serine 95, also determines the subcellular localization of the Kv4.2 potassium channel on plasma membranes [30] (Figure 1). Remarkably, this phosphorylation process does not modify the repressor activity of DREAM in the nucleus. In addition, DREAM can directly modulate several ligand- and voltage-dependent ionic channels, including N-methyl-D-aspartate receptors (NMDARs) [31,32] and voltage-dependent Ca^2+^ channels [33]. In particular, DREAM, upon the increase in [Ca^2+^]_i_ levels, binds to the NR1 subunit of the NMDAR and inhibits its currents [31,32]. This process might be interpreted as a negative feedback mechanism to reduce NMDAR-mediated Ca^2+^ influx. Another interesting mechanism of action of DREAM is the binding of important transcription factors, such as CREB, to counteract their activation. In particular, upon low levels of [Ca^2+^]_i_, DREAM reinforces its binding to CREB and prevents its activation by phosphorylation (Figure 2). Under these conditions, CREB is unable to recruit CREB-binding proteins (CBPs) and, thus, the CBP-mediated transactivation of genes provided with cAMP response elements (CREs) sequences is blocked [6] (Figure 2). By contrast, an increase in [Ca^2+^]_i_ impairs DREAM-dependent sequestration of CREB, thus allowing its phosphorylation and its transcriptional activity on target genes.

## 2. DREAM in CNS

### 2.1. Anatomical/Cellular Distribution

DREAM mRNA is found in embryos starting from day 10.5 post coitum, just after closure of the neural tube, indicating a potential role during development [34]. In fact, knock-out mice for DREAM show a transient reduction of astrocyte number during the early postnatal gliogenic period until the seventh day of postnatal life [35]. In adult mice DREAM mRNA is mainly expressed in the CNS, while a weak signal can be observed in testis, kidneys, and the spleen and thyroid [1,3]. As regards CNS, DREAM mRNA is expressed in almost all brain regions with peaks in the pyramidal layers of CA1, CA3 and granular layer of dentate gyrus of rat hippocampi. DREAM mRNA is also abundant in the piriform cortex, the anterior olfactory nucleus and in the anterior part of cerebellum. On the other hand, DREAM mRNA signals were not present in the Purkinje cells of cerebellum and in the molecular layer of cerebral cortex.

### 2.2. Physiological Roles of DREAM in CNS

DREAM is mainly expressed at the presynaptic level in neurons [36], where it participates in the regulation of glutamate release in the synaptic cleft [37] and plays a role in hippocampus-sensitive memory and synaptic plasticity [38,39,40]. In addition, DREAM binds to the NR1 subunit of the main receptor involved in synaptic plasticity, NMDAR, causing a reduced surface expression of this ligand-gated ion channel and, ultimately, a decrease in NMDAR-mediated currents [31]. Furthermore, DREAM transcriptionally downregulates another important player involved in neuronal [41] and oligodendrocyte differentiation [42], and spatial learning and memory [43], NCX3, by binding the DRE sequences on its gene promoter [10]. Indeed, the overexpression of a dominant positive construct of DREAM, named EFmDREAM, significantly downregulates mRNA and protein of NCX3 in neurons [10]. In contrast to its actions in neurons, DREAM was shown to have no effect on NCX3 promoter activity in the U87 glial cell line, supporting the hypothesis that the inhibitory mechanism of transcription is selective for neuronal cells [23]. Indeed, it has been reported that DREAM can also act as a transcriptional transactivator on the GFAP promoter during astrocyte differentiation after stimulation with pituitary adenylate cyclase-activating polypeptide (PACAP) [35]. Such apparent differences in the action of DREAM in glial and neuronal cells might be partially explained by an involvement of unknown transcription and/or epigenetic cofactors. On the other hand, it should also be considered that pleiotropic functions of DREAM, through the interaction with DRE sequences and/or with proteins in nucleus and in cytosol, are mainly influenced by free [Ca^2+^] that can rapidly change in subcellular compartments or in local microdomains with different kinetics following neuronal activation. In addition, some conditions including the presence of a different pattern of voltage-gate channels, and ionic pumps/exchangers, exposure to oxidative stress and induced neuronal plasticity, might further increase, or decrease, Ca^2+^ influx or clearance and, thus, the activity of DREAM. These highly variable and transient conditions that allow or do not allow for the Ca^2+^ binding of DREAM might prevail in some subcellular regions under some circumstances in a particular cell type. Furthermore, it should be underlined that, at the present, insufficient information is available regarding the concentration-binding curve of Ca^2+^ to DREAM and the consequent kinetics of DREAM activation/deactivation. Given the abovementioned complex variables, it is difficult to formulate hypotheses on the different transcriptional effects of DREAM on target genes in neuronal and non-neuronal cells.

## 3. DREAM and Stroke

Although the physiological roles of DREAM and its target gene are not completely clarified, a growing spectrum of studies correlates the activity of this Ca^2+^-sensor transcription factor with several neurological diseases including stroke. In fact, DREAM increases its expression levels in several models of stroke, including primary hippocampal neurons after 3 h of oxygen–glucose deprivation (OGD) where it enhances the secretase-induced cleavage of Notch, contributing to cell death under ischemia-like conditions. In addition, DREAM shows a small increase in the dentate gyrus 24 h after reperfusion and a dramatic increase throughout the hippocampus 72 h after reperfusion in a rat global ischemic model [44]. More important, DREAM increases in neurons of the peri-ischemic temporoparietal cortex region in a model of transient middle cerebral artery occlusion (tMCAO), where it downregulates the expression of the ncx3 gene [23]. Interestingly, this process involved another interesting mechanism of action of DREAM. Indeed, our research group showed that under these conditions, DREAM binds and drives two epigenetic enzymes, HDAC4 and HDAC5, on the DRE sequence of the ncx3 promoter [23]. Accordingly, both HDAC4 and HDAC5 are predominantly expressed in neurons [45] and increase following stroke injury, especially in neuronal nuclei [23]. Interestingly, this tight interplay forms a complex that epigenetically regulates the expression on the targeted gene by deacetylating histone proteins, thus condensing chromatin in this genomic region. This process is dependent on the presence of the DRE sequence on the ncx3 promoter, and each member of the complex, HDAC4, HDAC5 and DREAM, since it is completely lost when the DRE sequences are mutated or when one of either HDAC4 or HDAC5 are silenced. These data add a new function for DREAM and further expands the range of mechanisms by which this neuronal Ca^2+^ sensor controls gene expression. In fact, the existence of this crosstalk between protein–DNA and protein–protein interactions may also occur in other processes regulating gene expression driven by DREAM.

More important, the DREAM-dependent inhibition of ncx3 expression ultimately leads to the reduction of the neuroprotective effect exerted by this antiporter against stroke damage [25,46] (Figure 3). In fact, the pharmacological inhibition or genetic knock-down of DREAM show beneficial effects in experimental stroke, including tMCAO [47] and four vessel occlusion [44] models. On the other hand, opposite results were obtained with DREAM overexpression in an excitotoxicity neuronal model. In particular, when DREAM is overexpressed, its amino-acid region 21–40 located on the N-terminus binds to the C0 domain of the NMDA receptor subunit, NR1. This binding reduces NMDA-mediated currents, thus conferring neuroprotection against neuronal excitotoxicity. In addition, the different role of DREAM in glia and the contribution of these cells in stroke physiopathology should be considered.

## 4. DREAM in Neurodegenerative Diseases

Since DREAM serves as a Ca^2+^-sensor transcription factor in neurons, it can also participate in the progression of several neurodegenerative disorders, including Alzheimer’s (AD) and Huntington’s (HD) disease, and amyotrophic lateral sclerosis (ALS).

### 4.1. Amyotrophic Lateral Sclerosis (ALS)

ALS is the most rapidly progressing disease among neurological disorders [48]. Mean survival time for patients suffering of ALS spans between two to five years from the onset of symptoms [49,50]. Beside environmental neurotoxicants [51,52,53] that was postulated as responsible for ALS etiopathology, one of the altered molecular mechanisms that has been described in ALS is glutamate toxicity, which triggers an overstimulation of neuronal excitability, leading to an increase in [Ca^2+^]_i_ and, consequently, to an amplification of excitotoxic damage. In addition, the activity or cytosolic levels of several neuronal transcription factors seems to be altered under these conditions [54]. Interestingly, a strong positive signal of DREAM could be shown inside and around the nucleus of motor neurons and in astrocytes in some regions of the CNS, including the anterior horn of the spinal cord of ALS patients [55], thus suggesting the possible role of DREAM. Recent evidence has shown that the NCX3 gene, which is a target of DREAM, delays the neurodegenerative progression in a mouse model of ALS [56]. Indeed, a sub-toxic acute exposure to the cycad neurotoxin beta-methylamino-L-alanine (L-BMAA), a well-known compound mimicking ALS pathogenesis, exerts a preconditioning effect by increasing NCX3 expression in SOD1G93A mice [56]. DREAM expression and transcriptional activity has also been found to be increased in rat cortical neurons transfected with a sod1G93A construct, another model mimicking ALS, exposed to non-toxic concentration of thimerosal. Interestingly, neuronal death was reduced by the downregulation of DREAM [57].

The role of DREAM in ALS pathophysiology was also identified by other research groups demonstrating that its protein levels were significantly upregulated in motor neurons and astrocytes of spinal cord of transgenic SOD1G93A mice. DREAM immunostaining was mainly present in the nucleus compartment, whereas in the alive motor neurons, it was present near the membrane or in the cytoplasm. In support of these preclinical data, DREAM staining was also found in astrocytes present in the spinal cord and frontal cortex of ALS patients [55]. On the other hand, the calcium-dependent excitotoxicity in ALS could modulate the multifunctional nature of DREAM, strengthening its apoptotic way of action in both motor neurons and astrocytes. This modulation could act as an additional factor to increase neuronal damage. This direct crosstalk between astrocytes and motor neurons can become vulnerable under neurodegenerative conditions, and DREAM could act as an additional switch to enhance motor neuronal loss.

Supporting these results, recent genome-wide gene-set analysis experiments performed in the European descent ALS-control cohort of 9244 ALS cases compared to 12,795 healthy controls confirmed a DREAM dysregulation in ALS pathophysiology [58].

### 4.2. Alzheimer’s Disease

Alzheimer’s disease is a common and disabling neurological disorder that exponentially increases with age. The central hypothesis in the pathogenesis of AD is the activation of the amyloid cascade that leads to abnormal amyloid precipitates in the brain with neuronal dysfunction, the induction of tangles, neuronal death, and consequent dementia [59,60]. Presenilin 1 (PS1) and 2 (PS2) represent the catalytic constituent of the γ-secretase enzyme, which cleaves the amyloid precursor protein (APP) into Aβs of varying lengths. Mutations of either presenilin genes represent the main cause of familial forms of AD. Interestingly, DREAM was initially identified because it directly interacts with PS1 and PS2 in a Ca^2+^-independent manner, which regulates the levels of a proteolytic product of PS2 that, in turn, potentiates the decrease of endoplasmic reticulum Ca^2+^ release [29]. Based on this evidence, it is not surprising that DREAM might exert a role in AD, also considering that one of the target gene of DREAM, NCX3, is also involved in this neurodegenerative disease [61,62]. Furthermore, DREAM was found to be overexpressed in the activated astroglia surrounding β-amyloid (Aβ) plaques in the brain of Swedish mutant APP transgenic mice [63].

Furthermore, evidence that DREAM plays a role in AD includes: (i) transfection of increasing amounts of DREAM leading to an augmented formation of Aβ_42_ in HELA cells [64]; (ii) DREAM knock-out mice showing lower levels of Aβ_42_ peptide in the cerebellum, an area that physiologically expresses high levels of DREAM [38]; (iii) cortical and hippocampal neurons being exposed to Aβ_42_, upregulating DREAM expression, and causing apoptotic cell death [63]; (iv) DREAM being highly expressed in the brains of AD patients and of Tg2576 transgenic mice [63].

### 4.3. DREAM and Huntington’s Disease (HD)

Huntington’s disease is a neurodegenerative disease caused by the presence of CAG triplet in the huntingtin gene that ultimately causes alterations in [Ca^2+^]_i_ homeostasis with synaptic dysfunction and consequent neurodegeneration.

Interestingly, several lines of evidence have shown that DREAM is downregulated in (i) the striatum and hippocampus of R6/2 and R6/1 mice, two animal models reproducing HD; (ii) the striatal STHdhQ111/111 cell line, derived from knocking-in mice bearing 111 CAG repeats in the huntingtin gene; and (iii) in postmortem brain samples from HD patients. This decreased expression level of DREAM might be interpreted as a defense mechanism since the genetic knockdown or pharmacological inhibition of DREAM with repaglinide or with the most potent DREAM inhibitor, IQM-PC330, delays the onset of motor dysfunction and reduces striatal neuronal death in a mouse model of HD [65,66]. In addition, the downregulation of DREAM promotes the activating transcription factor 6 (ATF6)-dependent transcription as well as unfolded protein response (UPR)-dependent survival. These two mechanisms ameliorate cognition deficits in HD mice [65,67].

Thus, DREAM may represent a putative pharmacological target delaying the onset of cognitive impairment of HD [67].

### 4.4. DREAM and Neuropathic Pain

Although neuropathic pain cannot be classified as a neurodegenerative disorder, there are several conditions in which neuropathic pain is triggered by neurological diseases. In this regard, DREAM is highly expressed in the nucleus of sensory neurons where it can control the expression of several genes involved in nociception, including BDNF and prodynorphin genes. Notably, prodynorphin is post-translationally processed in opioid polypeptide hormones, such as endorphin [68], that potentiates antinociceptive processes. In addition, DREAM is expressed in several thalamic relay nuclei involved in somatosensory functions. Among these structures, the anterior, dorsal and ventral nuclear groups transmit impulses encoding pain as well as information from cutaneous receptors and from deep receptors in muscle and tendon. Based on this distribution in the CNS and a direct transcriptional inhibition of prodynorphin and BDNF genes, it is possible to hypothesize that DREAM could exert a role in nociception regulation. In fact, the loss of function of this Ca^2+^-sensor protein causes an increased basal level of prodynorphin expression and a reduced nociception signal triggered by thermal, mechanical and chemical stimuli in a mouse model of pain. Intriguingly, this analgesic effect was not accompanied by deficits in motor function, learning or memory, and immune and cardiac functions as it occurs in opioid treatments [14]. In addition, the activation of mGluR5 causes an increase in the protein stability of DREAM, contributing to the pronociceptive role of this metabotropic glutamate receptor [21]. For these reasons, the pharmacological inhibition of DREAM may represent an interesting new analgesic strategy for the treatment of pain because of the lack of typical side-effects of opioids. Moreover, formalin injections cause an increase in DREAM protein expression in the spinal cord of rat models of inflammatory pain [69], showing a possible feedback regulation of DREAM expression by inflammatory pain. In addition, more recently, it was found that the N-terminal 31–50 fragment of DREAM interacts with transient receptor potential vanilloid 1 (TRPV1) and reduces its surface localization in the rat dorsal root ganglia. This mechanism ultimately alleviates heat hyperalgesia and participates in the pain-relieving effect induced by peripheral inflammation [70]. In addition, the overexpression of a mutated form of DREAM that is lacking both Ca^2+^- and cAMP-dependent regulations reduced the expression of several genes related to pain in the spinal cord, including prodynorphin and BDNF, of transgenic mice. Under these conditions, the dominant active mutant of DREAM caused an increased basal level of hyperalgesia without change in A-type currents. Furthermore, these transgenic mice also showed a lack of enhancement of spinal reflexes and increased expression of BDNF following peripheral inflammation as compared to wild-type animals [12].

This growing evidence showing DREAM as a new pharmacological target for analgesia is of particular interest, also considering that neuropathic pain following nerve injuries represents a major challenge for an effective treatment due to the involvement of multiple and diverse mechanisms.

## 5. Conclusions

In the current review, we aimed to examine recent data on the pathophysiology of DREAM in the CNS with some projection to clinical applications. Our understanding of the molecular mechanisms by which DREAM is regulated and participates in the control of (1) gene expression by epigenetic and transcriptional mechanisms, and of (2) protein activity is still fragmented and incomplete; however, based on the available studies, we can conclude that DREAM exerts a neurodetrimental role by inhibiting the expression of neuroprotective genes or by directly binding to proteins that are useful for activating defense processes against pathological insults.

These findings drive home the concept that DREAM could represent an interesting pharmacological target to intervene in several neurological diseases.

## Data Availability

Not applicable.
